# Adding eptinezumab to brief patient education to treat chronic migraine and medication-overuse headache: Protocol for RESOLUTION—A phase 4, multinational, randomized, double-blind, placebo-controlled study

**DOI:** 10.3389/fneur.2023.1114654

**Published:** 2023-02-22

**Authors:** Rigmor H. Jensen, Henrik Winther Schytz, Cristina Tassorelli, Gisela M. Terwindt, Louise N. Carlsen, Aurélia Mittoux, Ole Østerberg, Richard B. Lipton, Stewart J. Tepper, Andrew Blumenfeld, Christofer Lundqvist

**Affiliations:** ^1^Department of Neurology, Danish Headache Center, Rigshospitalet-Glostrup, University of Copenhagen, Copenhagen, Denmark; ^2^Department of Brain and Behavioral Sciences, University of Pavia, Pavia, Italy; ^3^C. Mondino Foundation, Pavia, Italy; ^4^Department of Neurology, Leiden University Medical Centre, Leiden, Netherlands; ^5^H. Lundbeck, Copenhagen, Denmark; ^6^Department of Neurology, Albert Einstein College of Medicine, New York, NY, United States; ^7^Department of Neurology, Dartmouth Hitchcock Medical Center, Geisel School of Medicine at Dartmouth, Lebanon, NH, United States; ^8^The Los Angeles Headache Center, Los Angeles, CA, United States; ^9^Departments of Neurology and Health Services Research, Akershus University Hospital, Lørenskog, Norway; ^10^University of Oslo, Oslo, Norway

**Keywords:** migraine, preventive medicine, protocols and guidelines, medication-overuse headache, eptinezumab, Brief Educational Intervention

## Abstract

**Introduction:**

Migraine is a highly prevalent and disabling neurological disease. Excessive use of acute medications can lead to medication-overuse headache (MOH), occurring when a patient experiences an increasing number of headache and migraine days, despite taking greater amounts of acute medication. To treat MOH, a preventive migraine treatment and/or withdrawal of the overused medication(s) are advised. Brief Educational Intervention (BEI) has been shown to be an effective tool with promising results for MOH. Here, we report the design of a clinical trial that aims to evaluate the efficacy of eptinezumab, an anti-calcitonin gene-related peptide preventive migraine treatment, as an add-on to BEI for treatment of MOH in those with chronic migraine.

**Methods and analysis:**

RESOLUTION will be a phase 4, multi-national, randomized, double-blind, placebo-controlled study. This study will enroll approximately 570 participants with dual diagnoses of chronic migraine and MOH. Eligible patients will be randomly allocated to one of two treatment groups, BEI and eptinezumab (100 mg; *n* = 285) or BEI and placebo (*n* = 285), in a 1:1 ratio. The primary endpoint is the change from baseline in monthly migraine days over weeks 1–4. Secondary and exploratory endpoints will assess monthly migraine days over weeks 1–12, MOH remission, transition from chronic to episodic migraine, health-related quality of life, work productivity, and the safety and tolerability of eptinezumab in this patient population.

**Ethics and dissemination:**

This study will be conducted in accordance with good clinical practice. All patients will be fully informed about the study, including the risks and benefits of participation, and all participants will provide informed consent for participation in the trial and dissemination of results.

## 1. Introduction

For people under the age of 50 years, migraine is the leading cause of disability worldwide and is one of the most prevalent neurological diseases (1, 2). Though acute treatment is nearly universally used to relieve attacks, patients may experience decreased acute medication effectiveness over time, leading to a vicious cycle of increased headache frequency and disability despite taking increased amounts of acute medication. Without intervention this may lead to the development of chronic migraine (CM) and medication-overuse headache (MOH) (3–7). Medication overuse occurs in up to 50% of individuals with CM, triggering MOH in about 60 million people and making MOH one of the top 20 causes of disability worldwide (5, 8–10). A first step in reducing MOH is to educate patients and prescribers on the harmful effects of medication overuse (9, 11). At this point, a patient with CM and MOH may choose to stop or drastically reduce their intake of acute medications; however, this may lead to an initially worsened headache, and depending on the medication, withdrawal symptoms (8, 11). Additionally, a small subset of patients may require hospitalization to withdraw from the overused medication (12). Moreover, some patients subsequently redevelop MOH within year(s), a phenomenon known as relapse (8).

To ensure the effectiveness of acute medication withdrawal and prevent worsening of headaches during this withdrawal process, a combination of preventive migraine treatment and withdrawal of the overused medication(s) has been suggested as a plausible management strategy (13–15). Patient education is extremely important and universally recommended in patients with CM and MOH, because ultimately the patient is the one who decides when and how to treat each attack and how to implement necessary behavioral or lifestyle changes (16, 17). One form of patient education that has proven efficacy in the treatment of patients with MOH is Brief Educational Intervention (BEI) (9). A recent concealed double-blind randomized trial showed the effect of behavioral intervention during acute medication withdrawal (18).

BEI is safe, effective, and low in cost. It involves a short screening followed by individual feedback on how and why one should reduce the substance of concern; this approach has been proven to provide long-term results of medication reduction for patients suffering from MOH (11, 19). Similar approaches have been shown to be effective in the management of alcohol and drug addiction (20). However, for patients with complex MOH, BEI alone may not be enough to yield good outcomes (21). Coupling BEI with preventive migraine therapy may provide a patient with the most promising chance of recovering and breaking the cycle of MOH (15, 22). On average, BEI takes about 10 min to complete. Therefore, it should be feasible to adopt BEI in most headache clinics.

In previous studies, eptinezumab, an anti-calcitonin gene-related peptide (anti-CGRP) monoclonal antibody administered *via* intravenous (IV) infusion for migraine prevention in adults, has been shown to reduce the burden of migraine on patients as early as day one (23–26). An additional exploratory subgroup analysis of patients with CM and MOH in the PROMISE-2 study showed that eptinezumab was effective at reducing the number of migraine days for patients with this dual diagnosis (3). However, to date no studies have investigated if systematic application of BEI would improve outcomes in patients with MOH treated with eptinezumab. Here, we report the design of a randomized controlled trial to evaluate the efficacy of eptinezumab as an add-on to BEI for the prevention of migraine and the treatment of MOH, and in turn, the impact on health-related quality of life and work productivity.

## 2. Methods and analysis

### 2.1. Trial design and study setting

RESOLUTION is a phase 4, interventional, multi-national, multi-site, randomized, double-blind, parallel-group, placebo-controlled study. The study began 1 July 2022 and is anticipated for completion 30 May 2024. The target population is defined as patients with a dual diagnosis of CM and MOH according to the International Headache Society (IHS) International Classification of Headache Disorders, 3rd edition (ICHD-3) (4). Both CM and MOH diagnoses will be confirmed *via* prospectively collected information in a daily headache electronic diary (eDiary) during the screening period. Patients will be recruited from various countries (in Australia, North America, and Europe) and sites (~70 in total) during the planned recruitment period to ensure the required sample size is met. This study will be conducted in outpatient settings such as tertiary headache centers or neurology out-patient clinics. The RESOLUTION protocol described in this article is edition 1.1, with a date of 7 February 2022, and has been registered with EudraCT (2021-003049-40) and ClinicalTrials.gov (NCT05452239).

**Figure 1:**
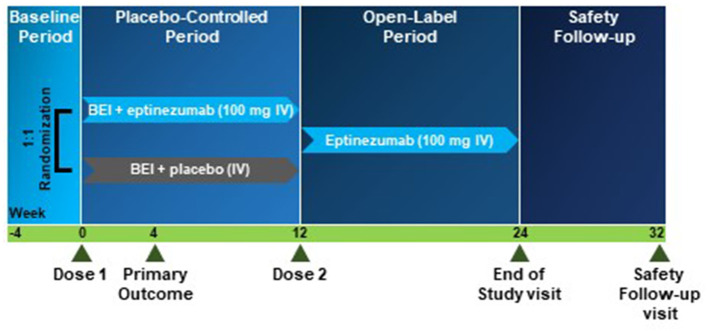
RESOLUTION study design. Study design for the randomized clinical trial included a baseline period (screening; 4 weeks), a placebo-controlled period (12 weeks) supplemented with Brief Educational Intervention (BEI), an open-label period (12 weeks), and a safety follow-up period (8 weeks). BEI, Brief Educational Intervention; IV, intravenous.

The total study duration will be approximately 36 weeks and includes a screening period (4 weeks), a placebo-controlled period (12 weeks), an open-label period (12 weeks), and a safety follow-up period (8 weeks; Figure 1). During the open-label period, all patients will receive eptinezumab (100 mg) to provide further relief, to gain exploratory data on the durability of a potential remission of MOH and CM, and to further assess the safety and tolerability of eptinezumab.

Patients are required to be on-site at the screening visit, at visits with study drug IV infusions (baseline visit and week 12 visit; IV infusion is administered over 30–45 min plus a post-infusion observation time of 1.5 h), and at the end-of-study visit (week 24). All other visits will be conducted as telephone or telemedicine visits. To support the assessment of endpoints, an eDiary will be filled in daily by each patient from the screening visit until either the end of study or the withdrawal visit. Adherence to eDiary compilation was monitored remotely.

### 2.2. Eligibility criteria

Patient selection is based on the main inclusion and exclusion criteria presented in Table 1 (for full inclusion and exclusion criteria please see Supplementary Table 1). Patients who meet all the inclusion criteria and none of the exclusion criteria are eligible to participate in this study.

**Table 1:** Main inclusion and exclusion criteria.

**Inclusion criteria prior to screening**
• The patient is aged ≥18 and ≤ 75 years of age • A dual diagnosis of CM and MOH as defined by ICHD-3 guidelines (4), with migraine onset at ≤ 50 years of age and a history of migraine for ≥12 months • ≥8 migraine days per month for each month within the past 3 months (per ICHD-3 guidelines (4)) • ≥15 headache days per month for each month within the past 3 months (per ICHD-3 guidelines (4)) • Failure with ≥1 preventive treatment within the last 5 years due to lack of efficacy* • Regular overuse of ≥1 drugs that can be taken for acute treatment of headache, for >3 months at a level that meets ICHD-3 criteria for MOH (4)
**Inclusion criteria during screening (based on information collected in the eDiary)**
• ≥15 to ≤ 26 headache days, of which ≥8 days were assessed as migraine days • Overuse of drugs that can be taken for acute headache treatment at a level that meets criteria for ICHD-3 MOH (4)
**Inclusion criteria after screening**
• Headache eDiary compliance for ≥24 of 28 days
**Main exclusion criteria**
• Previous anti-CGRP treatment failure including gepants for acute or preventive use • Confounding and clinically significant pain syndromes (i.e., fibromyalgia, chronic low back pain, and complex regional pain syndrome) • Severe psychiatric conditions whose symptoms are not controlled or who have not been adequately treated for ≥6 months prior to the Screening Visit • Clinically significant cardiovascular disease • Acute or active temporomandibular disorders • History or diagnosis of other headache disorders

^*^Lack of efficacy is defined as no clinically meaningful improvement at the locally recommended dose for ≥3 months.

CGRP, calcitonin gene-related peptide; CM, chronic migraine; eDiary, electronic diary; ICHD-3, International Classification of Headache Disorders, 3^rd^ edition; MOH, medication-overuse headache.

### 2.3. Interventions

#### 2.3.1. Brief Educational Intervention

**Figure 2:**
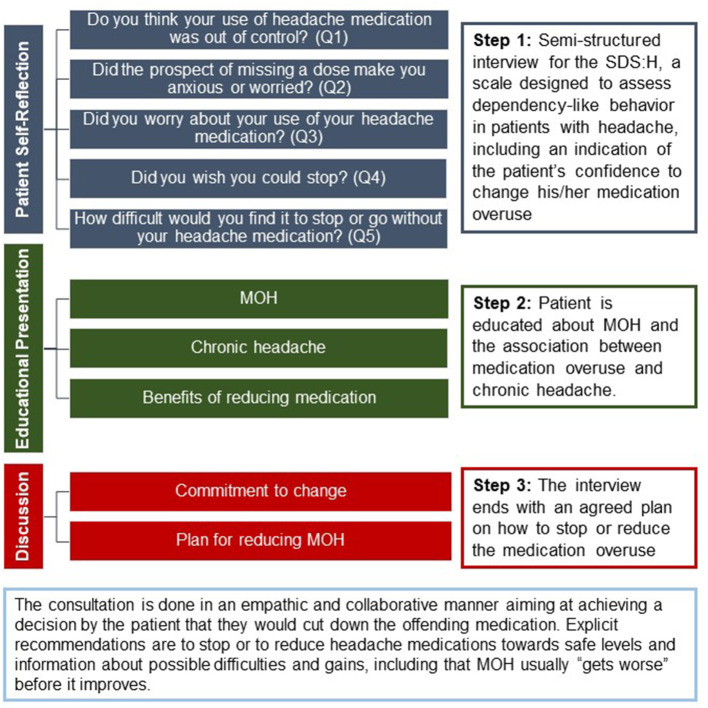
BEI: Assessing dependency-like behavior in patients with headache. The structure of Brief Educational Intervention (BEI) for treatment of MOH. MOH, medication-overuse headache; Q1–Q5, Questions 1–5; SDS:H, Severity-Dependence Scale for Headache.

All patients will receive BEI, administered by a trained clinician, at the baseline visit prior to study drug infusion to reflect real-world treatment scenarios. BEI is a 10-min, semi-structured educational conversation consisting of three components. First, patients are asked five questions adapted from the Severity Dependence Scale for Headache (SDS:H; Supplementary Figure 1) (27), including an indication of a patient's confidence to stop their medication overuse (Figure 2). Then, patients receive a short, structured presentation with information about MOH and the association between medication overuse and chronic headache. Topics include acute medication overuse, its side effects and pitfalls for migraine chronification, guidance for overcoming migraine chronification, and importantly, information that the headache may worsen initially before improvement occurs (i.e., “rebound headache”). Finally, BEI ends with a discussion on how to stop medication overuse and an agreed-upon plan for reducing acute medication use. At the baseline visit, patients will be advised to stop (or to limit the use of) their medications for acute and/or symptomatic treatment of headache (i.e., paracetamol/acetaminophen, nonsteroidal anti-inflammatory drugs, triptans, ergotamine, opioids, and/or combination analgesics). The discontinuation should be done abruptly. However, depending on the nature of the overused medication, the investigator can decide exceptionally to taper down instead. Patients are not excluded if they have used analgesics, but frequency of use will be recorded in the headache eDiary. Furthermore, the use of barbiturates and/or opioid analgesics are allowed provided a stable regimen of ≤ 4 days/month has been maintained for ≥12 weeks prior to screening.

#### 2.3.2. Study drug

Patients will be randomized to either BEI and eptinezumab 100 mg or BEI and placebo. Eptinezumab 100 mg will be dispensed as 1 single-use vial of 100 mg/mL (1 mL/vial) concentrate for solution for infusion, which is added to 100 mL of 0.9% normal saline, to be administered intravenously. Placebo will be dispensed as 100 mL of 0.9% normal saline, to be administered intravenously. Study drug is to be administered over a period of 30 min (up to 45 min as needed) by the blinded investigator or designee.

#### 2.3.3. Actigraphy assessments

To investigate the efficacy of eptinezumab as an add-on to BEI on daily physical activity and sleep, at certain sites consenting patients will have the option to participate in actigraphy assessments using a digital device for 24 h/day over the screening and placebo-controlled periods. Actigraphy is a non-invasive way of monitoring activity and sleep and is recorded using a wrist-worn device (EmbracePlus; Empatica, Boston, MA, US) that continuously records physiological data using an accelerometer, an electrodermal activity sensor, and a peripheral temperature sensor. The following actigraphy parameters will be derived to capture physical activity and sleep: movement intensity, rest (measured as minutes per 24 h in the rest epoch state [range 0–300: 0 = wake epoch; 101 = rest epoch; 102 = turn and toss epoch; 300 = rest interruption epoch]), total sleep time (the total time identified as sleep, per night, in minutes), wake after sleep onset (the amount of time spent awake after the sleep onset), sleep efficiency (the percentage of time asleep within the time-in-bed period), and sleep onset latency (the time from the start of the time-in-bed period to the actual sleep onset). Prior to use, a guide with details on how to use the device will be provided to the patient.

### 2.4. Randomisation

Prior to randomisation, the investigator will review the data in an eDiary eligibility report to determine if the eligibility criteria are fulfilled. Eligible patients will then be allocated *via* a randomisation system to one of the two treatment groups (1:1 ratio): BEI and eptinezumab 100 mg or BEI and placebo. The placebo group will be included to represent supportive care in the absence of pharmacotherapy. Each patient will be assigned a screening number, and that number will be used to identify the patient throughout the study. Randomisation of the patient will be performed by the interactive response technology (IRT) system and stratified by country and number of previous preventive treatment failures ( ≤ 2; >2) occurring ≤ 5 years prior to the baseline visit. For this study, treatment failure is defined as treatment discontinuation due to lack of efficacy (no clinically meaningful improvement at the recommended or prescribed dose for ≥3 months), side effects, or general poor tolerability of the treatment. The IRT will allocate the patient to a treatment group and assign the patient a randomisation number in accordance with the specifications from the biostatistics team.

The pharmacist will have access to the unblinded information for the double-blind treatment for each patient. All other study staff and patients will be blinded to treatment. The investigator may only break the code if knowledge of the study drug is necessary to provide optimal treatment to the patient in an emergency. If possible, the investigator should consult the clinical research associate before breaking the code, or as soon as possible. If this occurs during a visit, the investigator must complete the visit as a withdrawal visit. Otherwise, the patient will be asked to attend a withdrawal visit as soon as possible and a safety follow-up visit 20 weeks after study drug administration.

### 2.5. Data collection and management

During the screening period, after the informed consent form is signed (Supplementary material 1), participant information will be recorded, including baseline demographic data, complete medical history, migraine and treatment history, vital signs including electrocardiogram (ECG) recording, pregnancy screening, routine blood work, and baseline efficacy data. Throughout the study, patients will undergo efficacy and safety assessments as outlined in Table 2.

**Table 2:** Study procedures and assessments timeline.

End of week^*^	**−4**	**0**	**4^†^**	**8^†^**	**12**	**16^†^**	**24**	**32^†^**	**WD**
		**Infusion**	**Primary outcome**		**Infusion**		**End of study**	**Safety follow-up**	**Withdrawal**
**Screening and baseline procedures and assessments**									
Baseline demographics	°								
C-SSRS	°								
Inclusion/exclusion criteria	°	°							
Randomisation		°							
**Efficacy assessments (eDiary and ePROs)**									
eDiary recording^‡^^§^	°	°	°	°	°	°	°		°
eDiary compliance check		°	°	°	°	°	°		
PGIC			°		°		°		°
MBS	°	°			°		°		°
HADS		°	°		°		°		°
Digital device (optional)^||^	°	°	°	°	°				°
**Pharmacoeconomic assessments ePROs** ^¶^									
HIT-6		°	°		°		°		°
mMIDAS		°	°		°		°		°
MSQ v2.1		°	°		°		°		°
EQ-5D-5L		°	°		°		°		°
HCRU		°	°		°		°		°
WPAI:M		°	°		°		°		°
TSQM-9		°	°		°		°		°
**Safety assessments**									
Adverse events		°	°	°	°	°	°	°	°
Vital Signs	°	°			°		°	°	°
**Other procedures**									
Brief Intervention (with SDS:H)		°							

°, the respective assessment or procedure will occur that week.

^*^All assessments may be completed over a maximum of two consecutive days except for ePROs.

^†^Decentralized visits; the patient will be contacted for eDiary compliance check, to ensure that the selected assessments have been completed and for collection of relevant information such as adverse events and concomitant medications.

^‡^eDiary assessments will be completed in the remote setting daily from screening to end of study visit.

^§^On the baseline visit and week 12 visit day, patients must complete the ePRO entries prior to infusion.

^||^At designated sites, the patients undergoing the additional actigraphy assessments must provide a signed digital device Informed Consent Form.

^¶^ePROs scheduled at the baseline visit and week 12 visit must be completed in the clinical site on the visit date and before the infusion. ePROs that are scheduled in alignment with a decentralized contact must be completed in a remote setting and can be completed on the day or within 3 days prior to the scheduled decentralized contact date.

C-SSRS, Columbia-Suicide Severity Rating Scale; eDiary, electronic diary; ePRO, electronic patient-reported outcome; HADS, Hospital Anxiety and Depression Scale; HCRU, health care resource utilization; HIT-6, 6-item Headache Impact Test; MBS, most bothersome symptom; mMIDAS, modified Migraine Disability Assessment; MSQ v2.1, Migraine-Specific Quality of Life Questionnaire Version 2.1; PGIC, Patient Global Impression of Change; SDS:H, Severity Dependence Scale for Headache; TSQM-9, Treatment Satisfaction Questionnaire for Medicine−9 items; WD, withdrawal date; WPAI:M, Work Productivity and Activity Impairment questionnaire, adapted for migraine.

Electronic case report forms (eCRFs) will be used to collect all data related to the study, except for the external data (i.e., safety laboratory test data, eDiary data, actigraphy, and electronic patient-reported outcomes [ePRO data]), which will be transferred by the vendor and kept in a secure designated storage area separate from the eCRF. The eCRFs use third-party software (Rave^®^) to capture data *via* an online system on a computer. When the investigator enters data in the eCRF (ideally during the visit or no more than 3 days thereafter), the data will be recorded electronically in a central database over encrypted lines, and all entries and modifications to the data will be logged in an audit trail. For the actigraphy assessment, the data will be recorded on the internal memory of the wearable wristband and sent to a paired application (Care App) *via* Bluetooth. Data from the application are transferred to a research portal (Care Portal) *via* an internet connection. As this is a phase 4 study, with an approved treatment, a data monitoring committee is not required.

### 2.6. Outcome measures

The primary endpoint is the change from baseline in monthly migraine days (MMDs) over weeks 1–4 and will be assessed based on eDiary data after 4 weeks of collection during the placebo-controlled period. Key secondary outcomes will assess migraine days, headache days, acute medication days, and fulfillment of ICHD-3 diagnostic criteria for CM and MOH. Additional secondary and exploratory endpoints will be assessed as summarized in Supplementary Table 2.

Primary and key secondary endpoints will be tested sequentially in the order shown in Supplementary Table 2. Only if one step demonstrates a statistically significant effect will the formal testing continue to the next step; a significance level of 0.05 will be used. Assessing the primary outcome of this study at week 4 has been chosen both as an early marker of the efficacy of eptinezumab with BEI and to demonstrate that this intervention has the potential to rapidly break the vicious cycle that leads to MOH, thereby immediately reducing acute medication use and patient burden.

### 2.7. Statistical methods

#### 2.7.1. Sample size considerations

It is assumed that the treatment effect of eptinezumab 100 mg compared to placebo in change from baseline in MMDs (weeks 1–4) will be −1.5 MMDs. In DEFINE3, the differences between treatment + withdrawal and withdrawal were −1.7 and −1.4 days, respectively, after 2 and 4 months (15) and in the PROMISE-2 trial, 2.0 days between eptinezumab and placebo in the CM + MOH population (3). The standard deviation (SD) is assumed to be 6.2, based on the averaged SD across treatment groups in the MOH subgroup of PROMISE-2 on the change from baseline in MMDs (weeks 1–4) (3). Based on the assumed effect size and SD, using a two-sided test on the 5% significance level, 270 patients per treatment group will provide 80% power for showing an effect on the primary endpoint. Assuming 5% of patients will not contribute data to the analysis, 285 patients randomized per treatment group, or 570 patients randomized in total, will be needed. Sample size re-assessment will be conducted on blinded data when approximately 70% of the patients have been randomized. The SD is estimated in the same model as the primary endpoint, except that all terms including treatment (main effect of treatment group and interaction between treatment group and month) are excluded. Based on this estimate, the sample size may be increased if the estimated SD exceeds the planned SD to ensure 80% power on the primary endpoint, but not decreased.

#### 2.7.2. Analysis groups

For data analyses, the following analysis sets will be used: all randomized patients (APRS), all patients in the APRS who received an infusion of the study drug in the placebo-controlled period (APTS), all patients in the APTS who had a valid baseline assessment and ≥1 valid post-baseline 4-week assessment of MMDs in weeks 1–12 (FAS), and all patients in the APRS who received an infusion of the study drug in the open-label period (APTS-OL). The FAS will be used for all efficacy analyses in the placebo-controlled period, the APTS will be used for all safety analyses in the placebo-controlled period, and the APTS-OL will be used for the safety and efficacy analysis of the open-label period.

#### 2.7.3. Efficacy endpoint analyses

The primary endpoint will be analyzed using a mixed model repeated measures (MMRM) with the number of MMDs at baseline as a continuous covariate and including treatment group, month (weeks 1–4), country, sex, age, and previous treatment failures ( ≤ 2, >2) as categorical variables. The model will assume an unstructured covariance matrix to model the within-patient variance. The statistical test will be based on the treatment contrast for change from baseline in MMDs (weeks 1–4).

All continuous key secondary endpoints addressing changes from baseline to weeks 1–4 will be analyzed using the same methodology as described for the primary analysis, except for continuous key secondary endpoints addressing changes from baseline to weeks 1–12 in which the test will be based on the estimated mean MMDs averaged over weeks 1–4, 5–8, and 9–12. Daily pain assessment data will be collected in the headache eDiary *via* the question “What was the worst pain intensity of this headache today?”. The pain intensity assessment is collected on a 3-point scale: Mild (score = 1), Moderate (score = 2) and Severe (score = 3).

For each day, the daily pain assessment score will be derived by averaging the worst pain intensity over all headaches of that day. The average daily pain score will be calculated using the daily pain assessments collected during weeks 1–2. Change from baseline in average daily pain will be analyzed using the analysis of covariance (ANCOVA) with the average daily pain at baseline as a covariate and including treatment group, country, and previous treatment failures, as categorical variables. Regarding the use of acute medication, supplementary analyses looking only at days where no acute medication was used or where patients reported that the acute medication was not successful (this would be a composite strategy) will be done. The binary key secondary endpoints will be analyzed using logistic regression with baseline MMDs as a covariate and treatment and previous treatment failures ( ≤ 2, >2) as categorical variables.

#### 2.7.4. Handling missing data

For any patients who do not complete their eDiary for 24 of the 28 days per each 4-week period, there will be missing data. It is expected that most missing eDiary data will be sporadic. However, to account for this, for each 28-day period, missing data from the eDiary will be imputed in the following way: if the number of days with observations, *n*, is ≥14 days, the MMDs for the 28-day interval will be calculated as the number of migraine days observed/n × 28 (prorated) and rounded to 2 decimals and if *n* < 14, the MMDs for the period will be set to missing.

In the primary analysis, missing data will be regarded as non-headache/non-migraine days. Sensitivity analyses with missing days imputed as headache-free and days as migraine, and vice-versa, will be performed. For missing data in the quality-of-life questionnaires, missing items will not be imputed. However, strategies will be provided for calculating sub-score or total score with missing individual scores separately in the statistical analysis plan, which will be prepared by Biostatistics at the contract research organization before the study is unblinded. No imputation will be taken for missing data in the wearable digital device during the study period.

## 3. Discussion

### 3.1. Strengths and limitations of the RESOLUTION trial design

None of the new anti-CGRP monoclonal antibodies have been investigated as add-on treatment to medication management or MOH withdrawal education. This randomized controlled trial will evaluate the use of an anti-CGRP monoclonal antibody, eptinezumab, as an add-on to BEI in the treatment of MOH in patients with CM. The results of this trial are of high clinical relevance when determining if BEI, aiming to eliminate medication overuse, would be enhanced by a preventive treatment. Eptinezumab has high bioavailability and reaches maximum plasma concentration within 30 min of administration (28, 29). Offering patients an effective preventive treatment, that in combination with BEI can ameliorate the negative effects of stopping acute medications, could terminate acute medication overuse and secure lasting relief from MOH.

In this study design, the primary outcome will be assessed at week 4, allowing efficacy results to be determined quickly, which greatly benefits this subset of patients by allowing them to reduce their acute medication use while simultaneously reducing the burden of disease. Additionally, this study not only assesses a change in migraine days as the primary outcome but will also assess whether eptinezumab with add-on BEI improves the quality of life for patients with a dual diagnosis of CM and MOH, who have been identified as having a greater disease-based burden than patients with CM alone (30).

This study is being performed across approximately 70 sites in a variety of geographical locations with varying cultural differences regarding physician practices, patient expectations, medication availability, and regulatory rules between participating countries and headache centers. This will allow insight into how BEI and preventive treatment may benefit patients with MOH and CM in different settings and will allow for additional *post hoc* analyses regarding MOH treatment globally.

There are potential limitations associated with this study design, which focuses on patients with MOH with underlying CM and is therefore not generalizable to MOH patients with other underlying primary or secondary headache disorders. Additionally, patients with previous anti-CGRP therapy failures and those who use barbiturates and/or opioid analgesics more than four times per month will be excluded from participation, as will be individuals with clinically significant cardiovascular disease or confounding pain syndromes. Therefore, the findings from this study may not be indicative of safety and efficacy in the general population of patients with these or other excluded conditions.

## 4. Ethics and dissemination

### 4.1. Research ethics approval and consent

This study will be conducted in accordance with the International Conference on Harmonization note for guidance on Good Clinical Practice only after the sponsor has received confirmation that the regulatory authorities have approved or confirmed notification of the study and that written approval of the protocol has been granted by the appropriate ethics committee or institutional review board. All patients will be fully informed about the study, including the risks and benefits of their participation in the study. A patient may withdraw from the study at any time, for any reason, specified or unspecified, and without penalty or loss of benefits to which the patient is otherwise entitled. No study-related procedures, including any screening procedures, may be performed before the investigator has obtained written informed consent from the patient. Patients and/or the public were not involved in the design, conduct, reporting, or dissemination plans of this research.

### 4.2. Confidentiality and dissemination

The data collected will be processed in accordance with the specifications outlined in the Danish Data Protection Act and the European Union legislation to ensure that requirements regarding personal data protection are met. If an external organization processes data on behalf of the sponsor, a contractual procedure will be signed between the sponsor or delegate and the external organization to ensure compliance with the above-mentioned legislation. The results will be submitted to ClinicalTrials.gov and EudraCT and actively disseminated through peer-reviewed journals, conference presentations, and social media.

### 4.3. Data sharing

In accordance with the European Federation of Pharmaceutical Industries and Associations and Pharmaceutical Research and Manufacturers of America's “Principles for Responsible Clinical Trial Data Sharing” guidelines, Lundbeck is committed to responsible sharing of clinical trial data in a manner that is consistent with safeguarding the privacy of patients, respecting the integrity of national regulatory systems, and protecting the intellectual property of the sponsor. The protection of intellectual property ensures continued research and innovation in the pharmaceutical industry. Deidentified data are available to those whose request has been reviewed and approved through an application submitted to https://www.lundbeck.com/global/our-science/clinical-data-sharing.

## Author contributions

Conceptualized the project and developed the protocol: CL, RJ, HS, LC, OØ, and AM. Drafting of the manuscript, revising it for intellectual content, and final approval of the completed manuscript: RJ, HS, CT, GT, LC, AM, OØ, RL, ST, AB, and CL. All authors contributed to the article and approved the submitted version.
