# Supplementary material for: Adding eptinezumab to brief patient education to treat chronic migraine and medication-overuse headache: Protocol for RESOLUTION—A phase 4, multinational, randomized, double-blind, placebo-controlled study
Source: Front Neurol. 2023 Feb 22;14:1114654. doi: 10.3389/fneur.2023.1114654 (PMC9994537; doi:10.3389/fneur.2023.1114654)
Supplement: Supplementary file 1 [file Table_1.DOCX]

**Supplementary Table 1. Full inclusion and exclusion criteria**

| **Inclusion criteria**   1. The patient can read and understand the Informed Consent Form. 2. The patient has signed the Informed Consent Form. 3. The patient is an outpatient. 4. The patient has adequate venous access for administration of the study drug. 5. The patient has a diagnosis of CM as defined by IHS ICHD-3 guidelines [4] confirmed at the screening visit. 6. The patient has a history of migraine onset of ≥12 months prior to the screening visit. 7. The patient has ≥8 migraine days per month for each month within the past 3 months prior to the screening visit. 8. The patient has a diagnosis of MOH as defined by IHS ICHD-3 guidelines [4] confirmed at the screening visit. 9. The patient has ≥15 headache days per month for each month within the past 3 months prior to the screening visit. 10. The patient has regular overuse of ≥1 drug that can be taken for acute treatment of headache, for >3 months prior to the screening visit. 11. The patient has ≥15 to ≤26 headache days, of which ≥8 days were assessed as migraine days during the screening period, based on prospectively collected information in the eDiary. 12. The patient overuses drugs that can be taken for acute treatment of headache during the screening period, based on prospectively collected information in the eDiary. 13. The patient has a history of treatment failure with ≥1 preventive treatment within the last 5 years prior to the screening visit due to lack of efficacy (no clinically meaningful improvement at the locally recommended dose for ≥3 months). 14. The patient has demonstrated compliance with the headache eDiary by entry of data for ≥24 of the 28 days following the screening visit. 15. The patient has had an onset of migraine diagnosis at ≤50 years of age. 16. The patient is aged ≥18 and ≤75 years at the screening visit. 17. The patient, if a woman, must*:  - have had her last natural menstruation ≥12 months prior to the screening visit and had a high FSH level in the postmenopausal range as per local thresholds to confirm a postmenopausal state in women not using hormonal contraception or hormonal replacement therapy, OR - have been surgically sterilised (including tubal ligation, tubal occlusion, and oophorectomy) prior to the screening visit, OR - have had a hysterectomy prior to the screening visit, OR - remain sexually abstinent, when this is in line with her preferred and usual lifestyle, OR - engage exclusively in same-sex relationships, OR - agree not to try to become pregnant during the study AND use ≥1 of the below adequate contraception:   - combined oral, intravaginal, or transdermal hormonal contraception   - progestogen-only oral, injectable, or implantable hormonal contraception   - intrauterine devices   - intrauterine hormone-releasing system   - male or female condom with or without spermicide   - cap, diaphragm, or sponge with spermicide   - vasectomised partner - The contraception must be used from the screening visit to ≥6 months after the last dose of the study drug.  1. The patient has provided a signed optional subset-specific Informed Consent Form for actigraphy assessments, if applicable. |
| --- |
| **Exclusion Criteria**   1. The patient has previously been enrolled in this study. 2. The patient has experienced failure on a previous preventive treatment targeting the CGRP pathway including gepants for acute or preventive use. 3. The patient has participated in a clinical study <30 days or has taken any investigational products within 5 plasma half-lives (whichever is longer) prior to the screening visit. 4. The patient is a member of the study personnel or of their immediate families or is a subordinate (or immediate family member of a subordinate) to any of the study personnel. 5. The patient is pregnant, planning to become pregnant, or breastfeeding. 6. The patient has a history of severe drug allergy or hypersensitivity or known hypersensitivity or intolerance to any of the study drugs or its/their excipients. 7. The patient has confounding and clinically significant pain syndromes (for example, fibromyalgia, chronic low back pain, and complex regional pain syndrome). 8. The patient has a diagnosis of acute or active temporomandibular disorders. 9. The patient has a history or diagnosis of chronic tension-type headache, hypnic headache, cluster headache, hemicrania continua, new daily persistent headache, or unusual migraine subtypes such as hemiplegic migraine (sporadic and familial), recurrent painful ophthalmoplegic neuropathy, migraine with brainstem aura, and migraine with neurological accompaniments that are not typical of migraine aura (diplopia, altered consciousness, or long duration). 10. The patient has psychosis, bipolar mania, dementia, or any other psychiatric conditions whose symptoms are not controlled or who has not been adequately treated for a minimum of 6 months prior to the screening visit. 11. The patient has a current diagnosis or history of substance or alcohol use disorder (DSM-5^®^ criteria) <24 months prior to the screening visit. 12. The patient has any other disorder for which the treatment takes priority over treatment of migraine or is likely to interfere with study treatment or impair treatment compliance. 13. The patient has a history of moderate or severe head trauma or other neurological disorder or systemic medical disease that is, in the investigator’s opinion, likely to affect CNS functioning. 14. The patient has a history of cancer, other than basal cell or Stage 1 squamous cell carcinoma of the skin, that has not been in remission for >5 years prior to the first dose of study drug. 15. The patient has a history of clinically significant cardiovascular disease including uncontrolled hypertension, vascular ischaemia, or thromboembolic events (for example, cerebrovascular accident, deep vein thrombosis, or pulmonary embolism). 16. The patient has or has had ≥1 of the following conditions that is/are considered clinically relevant in the context of the study: other neurological, pulmonary, hepatic, endocrinological, gastrointestinal, haematological, infectious, immunological, or ocular disorder. 17. The patient takes or has taken recent or concomitant medication that is disallowed or allowed with restrictions, or it is anticipated that the patient will require treatment with ≥one of these medications during the study. 18. The patient has ≥1 clinically significant out-of-range vital signs at the screening visit. 19. The patient has a BMI ≥39 kg/m^2^ at the Screening Visit. 20. The patient has tested positive for HIV, anti-HCV, or hepatitis B serology that is confirmed to have acute or chronic infection. 21. The patient has ≥1 clinical laboratory test values outside the reference range, based on the blood and urine samples taken at the screening visit, that are of potential risk to the patient’s safety, or the patient has, at the screening visit:     1. a serum creatinine value >1.5 times the upper limit of the reference range     2. a serum total bilirubin value >1.5 times the upper limit of the reference range     3. an ALT or AST value >2 times the upper limit of the reference range 22. The patient has at the screening visit an abnormal ECG that is in the investigator’s opinion clinically significant. 23. The patient has a QTcF >450 ms (for men) or >470 ms (for women) at the screening visit, as calculated by the ECG equipment and evaluated by the investigator. The ECG may be repeated if any of the values are out of range or abnormal. 24. The patient is, at the screening visit, at significant risk of suicide (defined, using the CSSRS, as the patient answering: “yes” to suicidal ideation questions 4 or 5 or answering: “yes” to suicidal behaviour within the past 12 months). 25. The patient has a disease or takes medication that could, in the investigator’s opinion, interfere with the assessments of safety, tolerability, or efficacy, or interfere with the conduct or interpretation of the study. 26. The patient is, in the investigator’s opinion, unlikely to comply with the protocol or is unsuitable for any reason. |

*Inclusion and exclusion criteria are intended to safeguard against pregnancies during the study and if any pregnancies were to take place additional parental consent will be asked to monitor child health until 1 month after birth

ALT, alanine aminotransferase; AST, aspartate transaminase; anti-HCV, anti-hepatitis C virus; BMI, body mass index; CGRP, calcitonin gene-related peptide; CM, chronic migraine; CNS, central nervous system; CSSRS, Columbia-Suicide Severity Rating Scale; DSM-5^®^, Diagnostic and Statistical Manual of Mental Disorders, 5th edition; ECG, electrocardiogram; eDiary, electronic diary; FSH, follicle-stimulating hormone; HIV, human immunodeficiency virus; IHS ICHD-3, International Headache Society International Classification of Headache Disorders 3^rd^ edition; MOH, medication-overuse headache; ms, millisecond; QTcF, heart-rate corrected QT interval using Fridericia’s correction formula.
