# Supplementary material for: Adding eptinezumab to brief patient education to treat chronic migraine and medication-overuse headache: Protocol for RESOLUTION—A phase 4, multinational, randomized, double-blind, placebo-controlled study
Source: Front Neurol. 2023 Feb 22;14:1114654. doi: 10.3389/fneur.2023.1114654 (PMC9994537; doi:10.3389/fneur.2023.1114654)
Supplement: Supplementary file 2 [file Table_2.DOCX]

**Supplementary Table 2. RESOLUTION efficacy endpoints**

| **Primary endpoint:**   - Change from baseline in the number of MMDs (weeks 1–4) |
| --- |
| **Key secondary endpoints:**   - Change from baseline in MMDs (weeks 1–12) - Change from baseline in MHDs (weeks 1–4) - Change from baseline in MHDs (weeks 1–12) - Not fulfilling the ICHD-3 diagnostic criteria for CM nor MOH (week 4) - Not fulfilling the ICHD-3 diagnostic criteria for CM nor MOH (week 12) - Change from baseline in average daily pain assessment score (weeks 1–2) - Change from baseline in monthly days with acute medication use (weeks 1–4) - Change from baseline in monthly days with acute medication use (weeks 1–12) |
| **Secondary endpoints:**   - Not fulfilling the ICHD-3 diagnostic criteria for CM (week 4, week 12) - Not fulfilling the ICHD-3 diagnostic criteria for MOH (week 4, week 12) - Change from baseline in MMDs with use of acute medication (weeks 1–12) - Change from baseline in monthly days with triptan or ergotamine medication use (weeks 1–12) - Change from baseline in monthly days with individual non-opioid analgesics or NSAID medication use (weeks 1–12) - Change from baseline in monthly days with combination non-opioid analgesics medication use (weeks 1–12) - Migraine on the day after dosing (day 1) - Response: ≥50% reduction from baseline in MMDs (weeks 1–4, weeks 1–12) - Response: ≥75% reduction from baseline in MMDs (weeks 1–4, weeks 1–12) - Response: ≥50% reduction from baseline in MHDs (weeks 1–4, weeks 1–12) - Response: ≥75% reduction from baseline in MHDs (weeks 1–4, weeks 1–12) - Change from baseline in rate of migraine attacks with severe pain intensity (weeks 1–4, weeks 1–12) - Change from baseline in rate of headache episodes with severe pain intensity (weeks 1–4, weeks 1–12) - PGIC score (week 4, week 12) - MBS score (week 12) - Change from baseline in the HIT-6 total score (week 4, week 12) - Change from baseline in the mMIDAS total score (week 4, week 12) - Change from baseline in the MSQ v2.1 sub-scores (Role Function-Restrictive, Role Function-Preventive, Emotional Function; week 4, week 12) - Change from baseline in the EQ-5D-5L VAS score (week 4, week 12) - Migraine-specific HCRU (baseline, week 12) - Change from baseline in the WPAI:M sub-scores (Absenteeism, Presenteeism, Work productivity loss, Activity impairment; week 12) - Change from baseline in HADS-depression, and anxiety subscale scores (week 4, week 12) - Change from baseline in TSQM-9 (week 4, week 12) - Change from baseline in the HIT-6 total score (week 24) - Change from baseline in the mMIDAS total score (week 24) - Change from baseline in the MSQ v2.1 sub-scores (week 24) - Change from baseline in the EQ-5D-5L VAS score (week 24) - Migraine-specific HCRU (week 24) - Change from baseline in the WPAI:M sub-scores (week 24) - Change from baseline in HADS-depression and anxiety subscale scores (week 24) - PGIC score (week 24) - MBS score (week 24) - Change from baseline in TSQM-9 (week 24) - Change from baseline in MMDs (week 24) - Change from baseline in MHDs (week 24) - Not fulfilling the ICHD-3 diagnostic criteria for CM nor MOH (week 24) - Change from baseline in monthly days with acute medication use (week 24) - Change from baseline in average Daily Pain assessment score (week 24) - Change from baseline in monthly days with triptan or ergotamine medication use (week 24) - Change from baseline in monthly days with individual non-opioid analgesics or NSAID medication use (week 24) |
| **Exploratory endpoints:**   - Complete withdrawal of acute headache medication (weeks 1–4, weeks 5–8, weeks 9–12) - Change from baseline in SDS:H score (week 12) - Change from baseline of passive registration of movement (actigraphy; average per 28 days; week 4, week 12)   - Minutes with light PA (11–50 a.U.)   - Minutes with moderate PA (51–100 a.U.)   - Minutes with intense PA (101–200 a.U.)   - Minutes in rest period (range 0–300, 101: rest epoch) - Change from baseline in sleep metrics assessment as assessed by actigraphy (average per 28 days; week 4, week 12)   - Total sleep time (minutes per night)   - Sleep efficiency (percentage per night)   - Wake after sleep onset (minutes per night)   - Sleep onset latency (minutes per night)   **All analyses will also be done by week; for example, change from baseline to week 1, baseline to week 2, and baseline to week 12.* |

a.U, arbitrary unit; CM, chronic migraine; HADS, Hospital Anxiety and Depression Scale; HCRU, health care resource utilisation; HIT-6, Headache Impact Test; ICHD-3, International Classification of Headache Disorders, 3^rd^ edition; MBS, most bothersome symptom; MHDs, monthly headache days; MMDs, monthly migraine days; mMIDAS, modified Migraine Disability Assessment; MOH, medication-overuse headache; MSQv2.1, Migraine-Specific Quality of Life Questionnaire Version 2.1; NSAID, nonsteroidal anti-inflammatory drug; PA, physical activity; PGIC, Patient Global Impression of Change; SDS:H, Severity Dependence Scale for Headache; TSQM-9, Treatment Satisfaction Questionnaire for Medicine–9 items; VAS, visual analogue scale; WPAI:M, Work Productivity and Activity Impairment questionnaire, adapted for migraine.
