# Supplementary material for: Adding eptinezumab to brief patient education to treat chronic migraine and medication-overuse headache: Protocol for RESOLUTION—A phase 4, multinational, randomized, double-blind, placebo-controlled study
Source: Front Neurol. 2023 Feb 22;14:1114654. doi: 10.3389/fneur.2023.1114654 (PMC9994537; doi:10.3389/fneur.2023.1114654)
Supplement: Supplementary file 3 [file Data_Sheet_1.PDF]

Severity of dependence scale (SDS)

Circle the answer that best applies to how you felt about your use of ________________ (headache medication) over the past month

1. Did you ever think your use of ____________ (headache medication) was out of control?

Never or almost never	*0*
Sometimes	*1*
Often	*2*
Always	*3*

2. Did the prospect of missing a dose make you very anxious or worried?

Never or almost never	*0*
Sometimes	*1*
Often	*2*
Always	*3*

3. How much did you worry about your use of the headache medication?

Not at all	*0*
A little	*1*
Often	*2*
Always or nearly always	*3*

4. Did you wish you could stop?

Never or almost never	*0*
Sometimes	*1*
Often	*2*
Always	*3*

5. How difficult would you find it to stop or go without ____________ (headache medication)?

Not difficult at all	*0*
Quite difficult	*1*
Very difficult	*2*
Impossible	*3*
