# Supplementary material for: Adding eptinezumab to brief patient education to treat chronic migraine and medication-overuse headache: Protocol for RESOLUTION—A phase 4, multinational, randomized, double-blind, placebo-controlled study
Source: Front Neurol. 2023 Feb 22;14:1114654. doi: 10.3389/fneur.2023.1114654 (PMC9994537; doi:10.3389/fneur.2023.1114654)
Supplement: Supplementary file 4 [file Data_Sheet_2.PDF]

Electronic Signature Page

Full Title

Short Title

20007A - Informed Consent Form - Master ICF v1.1

Study Number 20007A

The following persons have electronically signed this document

Server Date and Time	Signed By
14-Jan-2022 16:18:38	MEMN - MeToya Monroe
**Reason for Signature**	Correctness and Completeness
14-Jan-2022 16:27:18	AUAT - Aurelia Mittoux
**Reason for Signature**	Clinical Approval
14-Jan-2022 16:49:59	PUFA - Puneh Fazlalipour-Almajavan
**Reason for Signature**	Correctness and Completeness

INFORMATION SHEET AND CONSENT FORM

STUDY TITLE:	Interventional, randomized, double-blind, parallel-group, placebo-controlled study of add-on eptinezumab treatment to brief educational intervention for the preventive treatment of migraine in patients with dual diagnosis of migraine and Medication Overuse Headache
PROTOCOL NUMBER:	20007A
SPONSOR:	H. Lundbeck A/S (Lundbeck)<br>2500 Valby<br>Denmark
STUDY DOCTOR:	[Investigator Name]<br>[Site Address]<br>[Office Hours Tel]<br>[Out of Hours Tel]
ETHICS COMMITTEE or INSTITUTIONAL REVIEW BOARD:	[EC/IRB Name]<br>[EC/IRB Address]<br>[Office Hours Tel]

You are invited to be in a research study to see if a study drug will help to prevent your chronic migraines and medication overuse headaches. This form tells you about this study. Please read this with care and ask the study doctor or study staff all your questions. If you want to take part in this study, you will be asked to sign and date this form, after you read and understand it.

Taking part in this study is voluntary. This means that you can choose if you want to take part in the study. If you agree to participate, your health care or your rights will not change. You can leave the study at any time, for any reason without penalty or loss of benefits. If you do not want to join the study, you can talk to the study doctor about your health care.

H. Lundbeck A/S (Lundbeck) is the drug company that will run and pay for this study. The study doctor is paid by Lundbeck to run this study.

An [ethics committee/institutional review board] has reviewed and approved this study to make sure that your rights and welfare are protected after you join this study. This committee/board will watch over this study while you are in it.

INFORMATION ABOUT THIS STUDY

Why is this study being done?

You are asked to participate in this study because you have chronic migraine, and you are overusing acute medication for your headaches. You are not the only person with this problem. Acute headache medications are those you use when you have a headache. Migraine is one of the most common brain illness that people ask doctors for help with. Migraine is a disease that can causes disability and sometimes leads to using too much headache medicine and/or using your headache medicine too often.

Stopping your acute headache medication will probably be good for you but in the short term it usually causes your headaches to get worse. Stopping acute headache medication can also cause withdrawal symptoms such as nausea, vomiting, feeling faint or lightheaded (low blood pressure), fast heartbeat, trouble sleeping, and other problems. The overuse of acute headache medication can be controlled by learning how to stop or lower the amount of acute headache medication you take; this is called an educational intervention.

About 570 adults will take part in this study. This study will be run at over 70 study clinics in different parts of the world such as in the North America, Europe, and Australia.

This study is testing the medication eptinezumab which is a monoclonal antibody (a protein made in a lab for a specific purpose) to prevent migraine and cluster headaches. Eptinezumab is approved by the United States (US) Food and Drug Administration (FDA), the European Medicines Agency (EMA), and other countries by prescription to prevent migraines in adults. It is given as a an intravenous (IV) infusion. This means that a liquid medicine is slowly injected into a vein in your arm.

The goal of this study is to test how well of a brief educational intervention and an eptinezumab infusion works compared to a brief educational intervention and a placebo infusion (a liquid that looks like the study drug but does not have any medicine in it).

A brief educational intervention consists of a conversation between you and the study doctor or study staff that looks at how your headaches affect your life, explains how medication overuse headaches happen, and creates a plan to help you stop using too much acute headache medication or using it too often. You will be asked to record your headache symptoms every day in a diary and the effects of stopping your acute headache medication.

This study is also testing whether a brief educational intervention and eptinezumab help improve your health-related quality of life and work life. There are several questionnaires you will be asked to fill out before you get any study drug and each time you come to the study clinic for a visit or by using an electronic diary called an eDiary.

These questionnaires include questions about the symptoms that bother you the most (such as pain, nausea, vomiting), how often you need to go to the hospital or an urgent care center for your headaches, how your headaches affect your work and other activities, whether you are anxious or are depressed because of your headaches, and how much you depend on the medicines you take for your headaches.

The goal of this study is to see how well people with chronic migraine and medication overuse headache respond to preventative treatment with eptinezumab when it is associated with a brief educational intervention. The study is also planned to see if eptinezumab is safe (how any side effects happen to you) for people in the study.

From this point on, any references to the word "study drug" can mean eptinezumab or placebo.

What will happen during the study?

You will be in the study for up to 36 weeks or about 9 months and you will have to visit the study clinic 4 times and be contacted by phone for telephone visits 4 times.

The study has 4 periods or parts: Screening, Placebo-controlled, Open-label, and Safety Follow-up Periods. The Placebo-controlled Period is double-blinded. This means that you, the study doctor, and study staff will not know if you are getting eptinezumab or placebo. After 12 weeks, the study will be open label. This means that you, the study doctor, and the study staff will know that everyone is getting eptinezumab.

At the Screening Visit, you will be checked to see if this study is a good fit for you. You will be asked to read and sign this form before you have any study tests. If you want to be in this study, your study doctor will first check if you can take part in this study. This is called screening. Screening must be completed within 30 days before you get the first dose of study drug.

If you are eligible (fit) to be in the study, you will come back to the study clinic for the Placebo controlled Period. A computer will decide if you get eptinezumab or placebo by chance (like flipping a coin). Half of participants will get eptinezumab and the other half will get a placebo (a liquid with no medication). During this period, you will get an infusion of study drug and have phone calls 4 and 8 weeks later.

At Week 12, the Open-label Period starts, and everyone will get an infusion of eptinezumab, have a phone call at Week 16, and an End of Study Visit at Week 24.

The Safety Follow-up Period starts after the End of Study Visit and you will come back to the study clinic for a visit at Week 32.

You will still be able to take your acute headache medications during the study. You will be asked to record when you take your acute headache medication in the eDiary for the study doctor and study staff. Note that the standard of care and supportive treatment

regimen may vary for different countries; therefore, adapt the text accordingly while developing the country-specific ICF.

The list below shows what will happen to you during the study. If you do not know these tests or want to know more, please ask your study doctor to explain.

Throughout the study you will complete questionnaires to check your headache and migraine symptoms and their impact on your daily life:

- Patient Global Impression of Change (PGIC): to check the overall change in your headaches following treatment. It takes approximate 1 minute to complete the PGIC.

- Headache Impact Test (HIT-6): to check how headaches affect your life and your ability to function normally in daily life. It takes less than 5 minutes to complete the HIT-6.

- Modified Migraine Disability Assessment (mMIDAS): to ask you about any disability related to your migraines, their impact on your work/school life, family life, social life, and leisure, sports, or your outside activities. It takes about 5 to 10 minutes to complete the mMIDAS.

- Most Bothersome Symptom (MBS): to check on your migraine-related symptoms that bother you the most. It takes less than 5 minutes to complete the MBS.

- Hospital Anxiety and Depression Scale (HADS): this scale measures your level of depression and anxiety. It takes about 5 to 10 minutes to complete the HADS.

- Migraine-Specific Quality-of-Life Questionnaire Version 2.1 (MSQ v2.1): to check your quality of life related to migraines. It takes about 5 to 10 minutes to complete the MSQ.

- EuroQoL 5-Dimension 5-Level (EQ-5D-5L): to check your overall state of health. It takes less than 5 minutes to complete the EQ-5D-5L.

- Health Care Resource Utilization (HCRU): you will be asked migraine-specific questions about how often you see a doctor, emergency/urgent care visits, and hospital admissions. It takes about 5 minutes to complete the HCRU.

- Work Productivity and Activity Impairment: Migraine (WPAI:M): this questionnaire will ask you how your headaches affect your productivity at work and during other activities. It takes about 5 minutes to complete the WPAI:M.

- Treatment Satisfaction Questionnaire for Medicine – 9 Items (TSQM-9): this questionnaire asks how satisfied you are with the study drug. It takes about 5 minutes to complete the TSQM-9.

- Severity of Dependence Scale Adapted for Headache (SDS:H): A study staff member will ask you questions about your headache medications. This questionnaire will be completed during the brief educational intervention. It takes less than 5 minutes to complete the SDS:H.

A brief educational intervention is a guided educational conversation with the purpose on helping the patients to reduce their medication overuse headache. The brief educational intervention starts with the five questions from the SDS:H. You will then be shown a presentation either on a flip chart or using slides with information about medication overuse headaches and how they are related to your chronic headache.

The study staff will train you how to use an electronic diary called an eDiary. The eDiary is an app or application that you can download on your smartphone or tablet. If you do not have a smartphone or tablet the study clinic will let you use one during the study. You are asked to record your headache symptoms and any medications you take every day in the eDiary.

[Text to be adapted at the country/site level or removed entirely in case the country does not take part in the actigraphy sub-study]You will be asked if you would like to participate in a sleep and activity study using a small watch type of device called an actigraph. This type monitoring is called actigraphy. Actigraphy is a way to check on your activity and rest without using needles and wires, it is non-invasive. The actigraph is lightweight, and the information is collected without you doing anything. You will get an overview of your physical activity and sleep collected during the study, when the study ends. The actigraphy part of this study is an option for some clinics, not all clinics in this study will participate. This part of the study is voluntary (up to you).

Visit 1 (Screening Visit):

After you have reviewed and signed this informed consent form, the following tests will be done during this visit to see if you are eligible (fit) to take part in the study. This means having some tests, including taking blood and urine samples and answering questions and questionnaires. This visit is done only in the clinic and may take 3 or more hours.

During the Screening Visit, 19.2 mL (almost 4 tsps) of blood will be taken from you. Additional blood and urine samples may be collected if retesting is needed.

- You will be asked about your age, sex, and race [adapt per country].
- You will be asked about your migraine and headache diagnoses and history.
- Your health, social, psychiatric (mental or emotional illness), and neurologic (brain, nerves, and senses) history will be reviewed to make sure you can join the study.

- The medications (prescription and over-the counter), herbs, vitamins, minerals, supplements, and other treatments you use or have used will be reviewed before you join the study.

- Your height and weight will be measured while you're wearing light clothing and no shoes.

- You will have an overall physical exam and the study doctor will carefully check your nervous system such as how your eyes move and work, how you speak, think, walk, move your arms and legs, and interact with the study doctor.

- Your temperature, blood pressure, and pulse rate will be measured.

- You will have blood taken from a vein to check your overall health and give a urine sample. The blood tests include testing for hepatitis B and C, HIV, and a blood pregnancy test if you are a woman who can get pregnant. The urine tests will include tests for drugs and alcohol. The study doctor or study staff will tell you if the hepatitis B, hepatitis C, HIV, or pregnancy test results are positive and will give you information according to the normal clinical practice. The results of these tests must be negative in order for you to be in the study.

- You will have an electrocardiogram which is a recording of the electrical activity of your heart.

- Your mental and emotional health will be checked using the Columbia-Suicide Severity Rating Scale (C-SSRS). You will be asked questions to see if you have suicidal thoughts or thoughts of hurting yourself or others. There is no evidence that being in this study will increase your risk of suicide, but these questions need to be asked to study participants in studies involving migraine headaches. It takes about 5 to 10 minutes to ask the questions and for the study staff to score your results.

- You will be trained in how to use an electronic diary (eDiary). You are asked to record your headache symptoms and any medications you take every day in the eDiary. This information will be used to see if you are eligible (fit) to participate in the study.

- You will be trained on how to use the electronic patient reported outcomes questionnaires.

- You will fill out the most bothersome symptom (MBS) questionnaire in the eDiary.

- The study doctor will ask about your use of alcohol, tobacco, caffeine, and marijuana.

Visit 2 (Baseline and First study drug infusion)

During this visit, the study doctor will check again to make sure that this study is a good fit for you. This visit is done only in the clinic may take 3 or more hours. You will be assigned to a treatment group.

The following tests, questions, and questionnaires will be completed before you get the study drug:

- You will participate in a brief education intervention as described previously.
- The study doctor will check for any headache symptoms you have.
- The study doctor will check with you if you are completing the eDiary as you were shown, and you will be asked to keep completing the eDiary every day during the study.
- Before you get the study drug, your study doctor will remind you to record the last day of any headaches you recently had. This applies to any recent headaches that were ongoing or headaches that you didn't record in the eDiary.
- You will be asked to complete the following questionnaires about your migraine symptoms and their impact on your daily life and work (HIT-6, mMIDAS, WPAI:M), most bothersome symptom (MBS),your overall health status (MSQ 2.1, EQ-5D-5L), how often you need to see a doctor or hospital for your migraines (HCRU), your levels of anxiety and depression (HADS), and how satisfied you are with the study drug (TSQM-9).
- The study doctor will ask about your use of alcohol, tobacco, caffeine, and marijuana.
- If you are a woman who can get pregnant, you will have urine collected for a pregnancy test before you get the study drug. This test must be negative for you to get the study drug infusion.
- You will get an infusion of the study drug.

Visit 3 (Telephone Visit)

Visit 3 will happen 4 weeks after you get the study drug. This visit will be done as a telephone call or using a smartphone, tablet, or computer. The following questions and questionnaires will be asked during this visit.

- The study doctor will ask about any side effects you may have.

- The study doctor will ask about all of the medications you are taking and if any have changed.
- The study doctor will check with you if you are completing the eDiary as you were shown, and you will be asked to keep completing the eDiary every day during the study.
- The study doctor will ask about your use of alcohol, tobacco, caffeine, and marijuana.
- You will be asked to complete the following questionnaires about your migraine symptoms and their impact on your daily life and work (HIT-6, mMIDAS, WPAI:M), your overall health status (MSQ 2.1, EQ-5D-5L), how often you need to see a doctor or hospital for your migraines (HCRU), your levels of anxiety and depression (HADS), how satisfied you are with the study drug (TSQM-9), and how your migraines have changed since the start of the study (PGIC).

Visit 4 (Telephone Visit)

Visit 4 will happen 8 weeks after you get the study drug. This visit will be done as a televisit using a smartphone, tablet, or computer. The following questions and questionnaires will be asked during this visit.

- The study doctor will ask about any side effects that you may have.
- The study doctor will ask about all of the medications you are taking and if any have changed.
- The study doctor will check with you if you are completing the eDiary as you were shown, and you will be asked to keep completing the eDiary every day during the study.
- The study doctor will ask about your use of alcohol, tobacco, caffeine, and marijuana.

Visit 5 (Second study drug infusion):

This visit will happen 12 weeks after the first infusion of study drug. Everyone at this visit will get an infusion of eptinezumab at this visit. This visit will be done only in the clinic may take 2 or more hours. The following tests, questions, and questionnaires will be completed during this visit.

- Your temperature, blood pressure, and pulse rate will be measured.

- If you are a woman who can get pregnant, you will have urine collected for a pregnancy test before you get the study drug. This test must be negative for you to get the study drug infusion.

- The study doctor will ask about any side effects that you may have.

- The study doctor will ask about all of the medications you are taking and if any have changed.

- The study doctor will check with you if you are completing the eDiary as you were shown, and you will be asked to keep completing the eDiary every day during the study.

- You will be asked to complete the following questionnaires about your migraine symptoms and their impact on your daily life and work (HIT-6, mMIDAS, WPAI:M), most bothersome symptom (MBS), your overall health status (MSQ 2.1, EQ-5D-5L), how often you need to see a doctor or hospital for your migraines (HCRU), your levels of anxiety and depression (HADS), how satisfied you are with the study drug (TSQM-9), how your migraines have changed since the start of the study (PGIC), and answer questions about your use of headache medication (SDS).

- The study doctor will ask about your use of alcohol, tobacco, caffeine, and marijuana.

Visit 6 (Telephone visit)

Visit 6 will happen 4 weeks after the second infusion of the study drug. This visit will be done as a telephone call or using a smartphone, tablet, or computer. The following questions and questionnaires will be asked during this visit.

- The study doctor will ask about any side effects that you may have.

- The study doctor will ask about all of the medications you are taking and if any have changed.

- The study doctor will check with you if you are completing the eDiary as you were shown, and you will be asked to keep completing the eDiary every day during the study.

Visit 7 (End of Study Visit)

This visit will happen 12 weeks after the second infusion of study drug. This visit will be done in the clinic and may take 2 or more hours. The following tests, questions, and questionnaires will be completed during this visit.

- Your temperature, blood pressure, and pulse rate will be measured.
- If you are a woman who can get pregnant, you will have urine collected for a pregnancy test before you get the study drug. This test must be negative for you to get the study drug infusion.
- The study doctor will ask about any side effects that you may have.
- The study doctor will ask about all of the medications you are taking and if any have changed.
- You will be asked to complete the following questionnaires about your migraine symptoms and their impact on your daily life and work (HIT-6, mMIDAS, WPAI:M), most bothersome symptom (MBS), your overall health status (MSQ 2.1, EQ-5D-5L), how often you need to see a doctor or hospital for your migraines (HCRU), your levels of anxiety and depression (HADS), how satisfied you are with the study drug (TSQM-9), and how your migraines have changed since the start of the study (PGIC).

Visit 8 (Safety Follow-up):

This visit will happen 20 weeks after the second infusion of study drug. This visit will be done as a telephone call or using a smartphone, tablet, or computer. The following questions and questionnaires will be completed during this visit.

- The study doctor will ask about any side effects that you may have.
- The study doctor will ask about all of the medications you are taking and if any have changed.

Withdrawal Visit

If you withdraw (decide to stop getting the study drug) from the study, you will be asked to attend a Withdrawal Visit as soon as possible, unless you withdraw your consent (stop participating in the study completely). If possible, you are also asked to complete a Safety Follow-up Visit scheduled 12 weeks after your last infusion of study drug. Withdrawal visits can be done at the clinic or this can be a televisit. The following tests, questions, and questionnaires will be completed at the Withdrawal Visit.

- Your temperature, blood pressure, and pulse rate will be measured.
- If you are a woman who can get pregnant, you will have urine collected for a pregnancy test before you get the study drug. This test must be negative for you to get the study drug infusion.
- The study doctor will ask about any side effects you may have.

- The study doctor will ask about all of the medications you are taking and if any have changed.
- If you are using an electronic device given to you by the study doctor, you will return it at this visit.
- You will be asked to complete the following questionnaires about your migraine symptoms and their impact on your daily life and work (HIT-6, mMIDAS, WPAI:M), most bothersome symptom (MBS) your overall health status (MSQ 2.1, EQ-5D-5L), how often you need to see a doctor or hospital for your migraines (HCRU), your levels of anxiety and depression (HADS), how satisfied you are with the study drug (TSQM-9), and how your migraines have changed since the start of the study (PGIC).
- The study doctor will ask about your use of alcohol, tobacco, caffeine, and marijuana (if you withdraw during the placebo-controlled period).

Study Tests	Screening	First Study Drug Infusion			Second Study Drug Infusion			Safety Follow-up	Withdrawal
**Visit Number**	**1**	**2**	**3**	**4**	**5**	**6**	**7**	**8**	
**Type of Visit**	**Clinic**	**Clinic**	**Televisit/ Home**	**Televisit/ Home**	**Clinic**	**Televisit/ Home**	**Clinic**	**Televisit/ Home**	**Clinic or Televisit**
Sign the informed consents	√								
Questions about your age, sex, and race	√								
Questions about your history, social, medical, psychiatric, and related to the nervous system	√								
Collect previous medical records to verify your migraine history	√								
Collect your previous medical records to check the preventive medications that you have taken for your migraine.	√								
Questions about your recent medications, prescription and non-prescription	√	√	√	√	√	√	√	√	√
Questions about your use of alcohol, tobacco, caffeine, marijuana	√	√	√	√	√				√

Study Tests	Screening	First Study Drug Infusion			Second Study Drug Infusion			Safety Follow-up	Withdrawal
**Visit Number**	**1**	**2**	**3**	**4**	**5**	**6**	**7**	**8**	
**Type of Visit**	**Clinic**	**Clinic**	**Televisit/ Home**	**Televisit/ Home**	**Clinic**	**Televisit/ Home**	**Clinic**	**Televisit/ Home**	**Clinic or Televisit**
Physical examination, including height measurement	√								
Measure body temperature, pulse, blood pressure, breathing rate and weight	√	√			√		√		√
Collect your blood, if you are a woman who can have children	√								
Collect your blood sample	√								
Collect your urine sample to test alcohol and drug screening	√								
Perform an electrocardiogram – to measure your heart rhythm and activity	√								
Study drug intravenous infusion		√			√				
Participate in a brief educational intervention		√							
eDiary and questionnaire training	√								

Study Tests	Screening	First Study Drug Infusion			Second Study Drug Infusion			Safety Follow-up	Withdrawal
**Visit Number**	**1**	**2**	**3**	**4**	**5**	**6**	**7**	**8**	
**Type of Visit**	**Clinic**	**Clinic**	**Televisit/ Home**	**Televisit/ Home**	**Clinic**	**Televisit/ Home**	**Clinic**	**Televisit/ Home**	**Clinic or Televisit**
Complete eDiary daily	√	√	√	√	√	√	√		√
Complete electronic questionnaires	√	√	√	√	√	√	√		√
Wear actigraphy (optional) [update or remove at country level]	√	√	√	√	√				√
Check for any side effects		√	√	√	√	√	√	√	√
Have a urine pregnancy test (for women who can get pregnant)		√			√		√		√
Have an exit interview								√	

What happens to the samples collected from you?

Blood and urine samples will be taken from you in this study. Your samples will be sent to a central laboratory for testing:

Clinics	Lab Address
European clinics	PPD Laboratories<br>Cluster Park<br>Kleine Kloosterstraat 19<br>B-1932<br>Zaventem Belgium
North American clinics	PPD Laboratories<br>1 Tesseneer Drive<br>KY 41076-9167<br>United States
Australian clinics	PPD Laboratories<br>61 Science Park Rd<br>#02-12-15, 19/20, The Galen<br>Singapore Science Park II<br>Singapore 117525

Your samples will be identified by a code and will not show who you are.

Your blood samples will be safely stored for up to 15 years after the end of the study. The sponsor will tell the central laboratories when your biological samples may be destroyed. After this time, your blood samples will be destroyed unless you allow the use of remaining samples collected from you for future research. Anyone who works with your samples will keep the coded samples and the results private.

During and after the study, you have the right to have your blood samples destroyed if you contact your study doctor, as long as your samples are still coded and can be found. If you leave the study, your blood samples may not be destroyed. If you want your blood samples to be destroyed, you will have to tell your study doctor. All the blood samples and test data collected before you left the study will still be used for study purposes. After you leave this study, no new blood samples or test data will be taken

What is expected from you?

While you are in the study you have to:

- Follow the study rules.
- Come to all study visits.
- Agree to be contacted by phone or video.

- Do not take any other drugs or remedies unless the study doctor has allowed them first. This includes prescription and over-the-counter drugs.
- There are some medications that you should avoid taking while you are in the study. Your study doctor will talk with you about this.
- Tell the study doctor about any new treatment or drug you take during the study.
- Give correct information about your health history and current health.
- Tell the study doctor about any health problems you have during the study.
- Start recording the required information as directed by the study staff as soon as you have received the eDiary and have received training on how use it.
- Complete the eDiary every day, and questionnaires before all visits or at the visit.
- Bring the eDiary when you come in for the study clinic visits.
- Complete all questionnaires as truthfully as possible.
- If you are female and if you become pregnant, tell your study doctor as soon as you know. For additional information, please see section on “Are there any reproductive risks?” below.
- Agree to not post about or discuss the study on social media.
- Be in touch with your study doctor or study staff and tell them if you have a change in your contact details or if you no longer wish to be in the study.
- Agree to not take part in any other study for at least 30 days before starting the study or during the study.

What will happen at the end of the study or if you stop your participation early?

The study doctor will contact you when the study is close to the end to help you with any questions you may have. You will also be asked to keep the eDiary updated, as it will be closed (you will not be able to use it anymore) at your final study visit. You may be able to get a prescription for the study drug after the study is over. Your study doctor will discuss your future health care choices with you after your participation in the study is finished.

Your study doctor and/or the sponsor may learn new facts during the study. You will be told about these new facts. You can then decide if you want to still be in the study. If you

leave the study, there will be no penalty and you will not lose any benefits you are entitled to. Leaving the study will not affect the quality of the health care you are given.

The study doctor may stop the study drug or end your taking part in this study for any of the following reasons:

- Staying in the study would be harmful for you.
- You did not follow instructions about what to do in the study.
- The study is cancelled.
- You have withdrawn your consent for participation in this study.
- You have a risk of harming yourself.
- You have any reaction to the study drug infusion.
- You are a female participant who became pregnant during this study.

The study doctor will tell you the reason(s) why you should stop being in the study.

BENEFITS AND RISKS

Are there any possible benefits of being in the study?

Eptinezumab has been tested in more than 2500 patients with migraine and has to be shown to work well. Taking part in this study may or may not help to treat your migraine. Your health could improve, stay the same, or get worse. The data we get from you during this study may help doctors learn more about the study drug and your disease and this may help future patients.

What are the potential risks and discomforts?

All medications can cause effects that are not wanted. These are called side effects. You may have no, or some, side effects, and they may be mild, moderate, or severe.

If you have any side effects, or are worried about them, always talk with your study doctor or study staff. Your study doctor and study staff will also be checking you for side effects. Your study doctor will discuss the best way to manage any side effects with you. The treatment of side effects will depend on the type and how severe side effect is. If a severe side effect or reaction happens, your study doctor may need to stop the study drug.

If, during study screening or participation, a previously unknown medical condition is discovered, the study doctor will check whether you need a referral to another doctor.

All medications can cause effects that are not wanted. These are called side effects. People who got the study drug in the past most often reported the following side effects:

- Redness and swelling of the inside of the nose and back of the throat (nasopharyngitis) was most often seen after the first infusion of the study drug. The number of people with this problem went down after the first dose and remained about the same.

- Hypersensitivity reactions, which included swelling (angioedema), itching (urticaria), facial flushing, and rash. Most hypersensitivity reactions happened during the infusion and were not serious, but often led to discontinuation or required treatment. Serious hypersensitivity reactions may happen. If you have a hypersensitivity reaction you may have to stop the study drug.

- Tiredness (fatigue) was most often seen during and after the first infusion. Following the first week and with the next infusions, the number of people with fatigue went down.

If any of these problems happen to you, tell your study doctor. There are some side effects that may not be known yet. If you notice any side effects that are not mentioned here, please tell your study doctor.

Risks from study procedures:

- To give the study drug, an IV catheter is inserted into your vein and kept there for about 30 to 45 minutes.

- There are potential risks associated with inserting the IV catheter into a vein and giving the study drug through this catheter. Some people may have nausea, anxiety, feeling faint, or some temporary discomfort during the catheter placement.

- You may have pain, bleeding at the insertion location, or bleeding under the skin causing a bruise where blood is drawn, or the catheter is inserted.

- There is a possibility that the insertion location could get infected, with swelling, redness, and pain.

- Possible side effects from blood draws include feeling faint, redness and swelling of the vein, pain, and bruising or bleeding at the clinic of where blood is drawn. These normally disappear a few days afterwards.

- It is rare but possible to have a symptom of serious infection of the bloodstream or heart valves, or symptoms of a blood clot in the lungs. If these rare but serious conditions occur, you would have to go to the hospital for treatment.

Blood pressure measurement

- You may have some discomfort as the cuff inflates and squeezes your arm, but it should only last a few seconds. Sometimes there are tiny red spots that appear after the test, just below the location of the cuff; they should be painless.

Electrocardiogram

- The electrocardiogram records the electrical activity of your heart. The sticky pads used may be cold when applied and sometimes cause some discomfort (slight redness or itching). If there is hair in the area where patches need to be applied, this area will be shaved in order to complete the electrocardiogram. Shaving may cause irritation.

Are there any reproductive risks?

Women who are **pregnant, breast-feeding or planning to become pregnant** during the study will not be allowed to take part in the study. Women who could become pregnant must have a pregnancy test to rule out pregnancy before they start taking the study drug. After joining the study, women must report immediately to the study clinic if they suspect they are pregnant during the study.

Women able to get pregnant and who are having sex with a man, must use birth control during the study. Birth control methods that can be used while in this study include the following:

- You have been surgically sterilized including tubal ligation, tubal occlusion, and oophorectomy (having your ovaries removed) before the Screening Visit.
- You have had a hysterectomy prior to the Screening Visit
- You do not have sex with men (sexual abstinence), when this is your preferred and usual lifestyle
- You are exclusively in a same-sex relationship
- You use one of the following methods of birth control:
    - A combined pill, intravaginal, or transdermal hormonal contraception
    - Progestogen-only pill, injection, or implant (under the skin) birth control

- An IUD (intrauterine devices)
- An intrauterine hormone-releasing system
- A male or female condom with or without spermicide
- A cervical cap, diaphragm, or sponge with spermicide
- You have a partner who had a vasectomy

You must use birth control from the Screening Visit to at least 6 months after the last infusion of the study drug. If you use hormonal birth control you must be on the same medication for at least 12 weeks prior to the Screening Visit.

You must discuss with the study doctor the type of birth control method that you use before you begin the study. The study doctor must approve the method you use before you can enter the study.

If you become pregnant during the study, you must tell the study doctor immediately, and you will have to stop getting the study drug. The study doctor will advise you about your health care and will ask about your pregnancy and its outcome.

Men, currently there is no information that eptinezumab affects your reproductive system (such as your sperm or semen). The amount of eptinezumab in your semen is very low and should not affect your female partner(s). There is no requirement that you use birth control. Any pregnancy(ies) with your female partner(s), will not be followed for this study.

Are there any other treatments?

If you decide not to participate in this study, you may receive the standard treatment(s) for migraine. Your study doctor will explain the risks and benefits of these other treatments before you decide if you want to take part in the study.

COSTS AND COMPENSATION FOR STUDY PARTICIPATION

Are there any costs if you decide to take part in the study?

Taking part in this study will not cost you anything. You will not be charged for the study drug or any of the tests that are part of the study. The drug company will not pay for doctor visits or other treatments or tests that are not part of this study. This means that you, your insurance company, or your government's health plan may have to pay for these.

Your travel expenses to and from the study clinic may be paid for after you spend the money. Talk to your study doctor about any costs that might not be paid by the drug

company. Adapt accordingly per the country and study/Client-specific requirements in the country-level ICFs.

Will you receive any payment if you take part in the study?

You will not receive payment for taking part in this study. The text may also have to be customized if needed at the country/site level during the country-/site-specific ICF preparation.

Will you receive compensation for injury resulting from the study?

You should inform the study doctor as soon as you feel that you have had an illness or injury related to the study, so that you can get proper health care. If you are injured as a result of taking part in this study, you will be compensated according to the local law. Adapt accordingly per the country and study/Client-specific requirements in the country-level ICFs. Your study doctor will explain more about this to you.

It is important that you be careful to follow all the instructions given by the study doctor and study staff about this study.

By signing this form, you are not giving up your legal rights and are not releasing the study doctor or drug company from their legal and professional responsibilities.

CONFIDENTIALITY AND AUTHORIZATION TO COLLECT, USE, SHARE AND DISCLOSE PERSONAL HEALTH DATA

What happens to the data collected about you?

1. Which law applies for the processing of my personal data?

H. Lundbeck A/S (Lundbeck) is the sponsor of this study and is based in Denmark. Lundbeck will be using information from you and/or your medical records to run this study, and Lundbeck is the data controller for this study. This means that Lundbeck is responsible for looking after your information that is collected by your study doctor for this study and for using it properly and in accordance with the General Data Protection Regulation (GDPR) and the Danish Data Protection Act.

2. Why will my personal data be processed?

The purpose of collecting and processing your data is to do clinical research (human studies or scientific research) in the form of a scientific evaluation of a medicine to prevent migraine. As described in this *informed consent form,* this also includes archiving (storing) the data and collecting and processing safety (side effect) information obtained in the study.

3. Is Lundbeck allowed to process my personal data?

Lundbeck will collect, process, and analyze your personal data for this study and future scientific research. Lundbeck has a legitimate interest in conducting scientific research and processing your personal data in order to develop safe and efficient medicine. Your safety information such as any side effect(s) you may have will be processed, and your data stored (archived) to ensure high standards of quality and safety of the study drug.

4. How will my personal data be collected?

Your study doctor will collect information either directly from you and/or from your medical records, from your general practitioner, or through analyse of your blood and urine samples and in accordance with Lundbeck's instructions.

As stated in this *informed consent form*, your participation in the study is voluntary. However, if you decide to participate, you must follow the instructions provided by your study doctor. Not following your study doctor's instructions will lead to you leaving the study.

5. What kind of data will be collected?

The personal data collected for the study and processed by Lundbeck through your study doctor will be demographic data (for example, year of birth, sex, body weight), health data (for example, medical history, diagnosis of disease and severity, blood pressure, electrocardiogram results), and biological samples and results of analyses of them (for example, laboratory data). The categories of data to be collected and processed are described further in this *informed consent form*.

6. Who will be able to know my identity?

Your name, contact information, or any other information that allows your direct identification will be replaced by a code number at the study clinic.

Your study doctor will keep this information confidential and will not pass this information to Lundbeck. Your study doctor will use this information as needed, to contact you about the study, and make sure that important information about the study is recorded for your care. However, the following groups may also be able to identify you by name when they access your personal data at your study doctor's practice to check the accuracy of the data: selected Lundbeck employees, staff from the company that helps Lundbeck run this study, and national health authorities.

7. What will happen to your personal data outside my study doctor's practice?

Study data collected by your study doctor through interviews or examinations will be processed via secure systems provided by Lundbeck or Lundbeck's data processors (companies that help run this study), and all the data will only be identified by the assigned code number, so your information will not be directly identifiable by Lundbeck or Lundbeck's data processors. In some cases, Lundbeck may also anonymise your data (take out any information which can identify you) if we share the data with other scientists.

8. Who are the categories of recipients of my data outside my study doctor's practice?

Your data will be shared with the following categories of recipients:

- Your data will be processed on Lundbeck's behalf by a company helping to run the study (Pharmaceutical Product Development, also called PPD).
- Your blood samples, measurements of your blood pressure, electrocardiogram results, and other categories of data/assessments will be processed on Lundbeck's behalf and instructions by professional institutions/organizations (for example, central laboratories).
- After your coded data are entered into Lundbeck's systems, Lundbeck may share the study data, including your coded data, with the relevant ethics committee/ institutional review board, national health authorities, consultants, IT service providers, and research and collaboration partners, such as:
  – Electronic diary and electronic questionnaire provider: Clario (1818 Market Street, Suite 1000, Philadelphia, PA 19103, USA).
  – Actigraphy device provider: Empatica Inc., (45 Bromfield St, Suite 901, Boston, MA 02108, USA).
  – Your personal data will not be released to any other third party.

A list of the third parties involved in the study conduct and/or reporting can be provided upon request.

9. Will my data be shared with parties outside the EU/EEA?

Some countries outside the European Union (EU)/European Economic Area (EEA) may not have the same level of data protection as within the EU/EEA. If your data are transferred to countries outside the EU, this will be done in accordance with applicable legislation. If the recipient country is not a so-called safe country (see the list of countries on the EU Commissions' website https://ec.europa.eu/info/law/law-topic/data-protection/data-transfers-outside-eu/adequacy-protection-personal-data-non-eu-countries_en), Lundbeck will ensure that measures are in place to ensure an adequate level of data protection compared to that in the EU. For more information, please see

the EU-US Privacy Shield (https://www.privacyshield.gov/welcome) or the European Commission's Standard Contractual Clauses (https://ec.europa.eu/info/law/law-topic/data-protection/data-transfers-outside-eu/model-contracts-transfer-personal-data-third-countries_en).

Transfers made to ethics committees/institutional review boards or national health authorities in countries outside the EU will be made as the transfer is necessary for important reasons of public interest. Transfers made to other Lundbeck entities outside the EU will be based on Lundbeck Intra Group Agreements, which can be provided upon request.

10. How long will my personal data be stored?

Your data and the code list (connecting your personal information to the code number) will be stored securely by your study doctor for 25 years or longer if required by national laws. The coded personal data from all study participants will be stored in the secure systems held by Lundbeck for at least as long as required by applicable legislation regarding clinical research and medicinal products and as long as the data are considered to have scientific value.

11. Future scientific research

Lundbeck may also process and analyse the study data mentioned above under 'What kind of data will be collected for the purpose of future scientific research within Lundbeck's area of expertise (please refer to Lundbeck.com for more information). Your study doctor will not have access to data involved in Lundbeck's future scientific research.

Conducting future scientific research may mean that your coded data obtained as part of this study are at a later stage pooled (combined) with data from other studies. Further, your data may also be shared with Lundbeck's data processors, ethics committees/institutional review board, health authorities, and/or other researchers or collaboration partners, however, at this time, we cannot specify who the relevant research and collaboration partners may be or where they are located. Therefore, we cannot rule out that some of them may be located outside the EU where the level of data protection is not as good as within the EU.

13. Your rights

Subject to exceptions and restrictions set out in applicable legislation, you have the right to request access to your personal data, to object to the processing, to have your personal data corrected (rectified), deleted, or processing thereof restricted, and to data portability. However, your data are collected for scientific research, which is a highly regulated area and there are high standards for data reliability and accuracy. On this

basis, you should expect broad restrictions to your rights based on applicable legislation.

If Lundbeck process your personal data based on your consent you can withdraw your consent by contacting your study doctor who will coordinate with Lundbeck.

If you withdraw from the study, the data, including samples, already collected will be used.

If your rights in relation to your personal data have been violated, you have the right to file a complaint with the competent supervisory authority, such as the Danish Data Protection Agency, United State Food and Drug Administration, Medicines and Healthcare Products Regulatory Agency, or the supervisory authority in your home country.

As mentioned above, Lundbeck as sponsor only holds the coded data of study participants and cannot see whether a particular individual participated in a study or not. Lundbeck therefore recommends you contact your study doctor if you have any questions about the processing of your personal data. Your study doctor will direct your requests, as needed, to the sponsor by using your code number. This will ensure that your request will be dealt with in the most confidential way and your identity does not need to be revealed to the sponsor. If you feel that your study doctor is not able to address any question you have about the processing of your personal data and your rights, you can contact the sponsor's Data Protection Officer using the below contact details.

Contact details of Lundbeck:
H. Lundbeck A/S
Ottiliavej 9
2500 Valby
Denmark
Tel: +45 36 30 13 11

Data Protection Officer: Dataprivacy@lundbeck.com (support and advice in English and various European languages is available.)

What if you change your mind and do not want your data to be used or disclosed?

If you leave the study early, data obtained while you were in the study may still be kept with other data obtained as part of the study. Normally, no new data will be collected for the study unless you clearly agree to that. However, national health authorities require that you report side effects that you have even after you leave the study.

Will information about this study be publicly available?

A description of this clinical trial will be available at http://www.ClinicalTrials.gov, as required by U.S. Law. This website will not include information that can identify you. At most, the website will include a summary of the results. You can search this website at any time. Do not alter the wording of this paragraph. This requirement is based on U.S. regulation and it is relayed to all potential subjects during the consent process regardless if they are in the U.S. or not (U.S. CFR title 21 part 50.25).

A brief report of this study will be available at https://clinicaltrialsregister.eu. To be updated in the country specific ICF).In addition, the study will be available on the [country name] clinical trial registry. If required, the following statement may be included for non-English speaking countries during country-level ICF preparation: This website only shows data in English, but you can ask the study staff at any time and be given the information that is available to the public. These websites will not include data that can identify you. They will include a scientific report of the results of the study. You can search these websites at any time.

After this study is over, a brief report of the overall results will be prepared for the general public. The study results may also be shared with scientific journals and the scientific community. Whenever the results of the study are shared or published, your identity will remain private.

CONTACTS

Who can you contact with further questions?

If you have any questions about the study or if you feel that this study has caused you harm or injury, please contact the study doctor whose details are given on the first page of this form.

If you have any questions about your rights as a person taking part in this study, please contact the [ethics committee/institutional review board] that has reviewed and approved this study, also given on the first page of this form. There may be country-specific variations regarding the patient rights and contacting procedures. The text here should be customized accordingly with adequate details while preparing the country-specific ICF.

STATEMENT OF CONSENT

- I have read and understand the statements in this informed consent form.
- I have had the chance to ask questions, and I am satisfied with the answers given to me.
- I understand that this study may only be performed by collecting and using my health data. Therefore, by signing this form, I specifically give permission for my data to be checked, transferred, and processed as follows:
  - The authorized representatives of H. Lundbeck A/S (Lundbeck), the ethics committee/institutional review board, and inspectors for regulatory authorities may review my health data by directly accessing my health records.
  - Study data, including my coded health data, may be used, and shared for legitimate study and scientific purposes.
- I agree to take part in this study of my own free will.
- I understand that I and/or my legal representative will receive a copy of this signed and dated written informed consent form.

____

Printed Name of Participant, in full

____

Signature of Participant **Date (dd-Mmm-yyyy)**

- I have presented the study and answered the subject's questions.
- I will give the subject/legal representative a copy of this signed and dated informed consent form.

Printed Name of Person Obtaining Consent (Investigator/Delegate), in full

---

Signature of Person Obtaining Consent **Date (dd-Mmm-yyyy)**

If required,

Printed Name of Legally Authorized Representative or Legal Guardian (if subject is a minor), in full

---

Signature of Legally Authorized Representative or Legal Guardian (if subject is a minor) **Date (dd-Mmm-yyyy)**

If required,

Printed Name of Impartial Witness, in full

Signature of Witness **Date (dd-Mmm-yyyy)**

[Text to be adapted at the country/site level or removed entirely in case the country does not take part in the actigraphy substudy]

OPTIONAL CONSENT FOR ACTIGRAPHY PARTICIPATION

If you are willing to participate in the actigraphy part of this study, please check the appropriate box below and sign and date below.

By signing this part of the ICF, you confirm that you will allow collection of actigraphy information for this study.

☐ Yes, I agree that information from the actigraph may be collected, shared, and analyzed by Lundbeck.

☐ No, I do not agree that information from the actigraph may be collected, shared, and analyzed by Lundbeck. If you check this box, you will not be able to participate in the actigraph part of this study.

Printed Name of Participant, in full

Signature of Participant **Date (dd-Mmm-yyyy)**

If required,

Printed Name of Legally Authorized Representative or Legal Guardian (if subject is a minor), in full

---

Signature of Legally Authorized Representative or Legal Guardian (if subject is a minor) **Date (dd-Mmm-yyyy)**

If required,

Printed Name of Impartial Witness, in full

Signature of Witness **Date (dd-Mmm-yyyy)**
